# Tailoring Li–Al–O Interphases in Garnet-Type Solid-State Electrolytes via Powder Atomic Layer Deposition

**DOI:** 10.1021/acsami.5c23254

**Published:** 2026-03-25

**Authors:** Michael K. Steinhoff, Anna Domgans, Jehad Ahmed, Roland Schierholz, Davis Thomas Daniel, Nabi Aghdassi, Shicheng Yu, Hermann Tempel, Rüdiger-A. Eichel

**Affiliations:** † Institute of Energy Technologies—Fundamental Electrochemistry (IET-1), Forschungszentrum Jülich, 52428 Jülich, Germany; ‡ Material and Processes of Electrochemical Energy Storage and Conversion, 9165 RWTH Aachen University, 52074 Aachen, Germany

**Keywords:** surface modification, ALD powder coatings, Li–Al–O interphase, multiphase structure, garnet-type electrolyte, LLZTO

## Abstract

Garnet-type Li_6.4_La_3_Zr_1.4_Ta_0.6_O_12_ (LLZTO) solid-state electrolyte (SSE) faces challenges such as high interfacial resistance and lithium dendrite propagation. Meanwhile, atomic layer deposition (ALD) offers precise control over surface chemistry and nanoscale interfacial structures, enabling critical advancements in SSE design. Here, we investigate the influence of Al_2_O_3_ ALD powder coatings on LLZTO, with emphasis on structural evolution, chemical interdiffusion, and electrochemical performance. ^27^Al magic angle spinning NMR, XPS, and STEM measurements confirm lithium diffusion during ALD, forming a compositionally graded, nanocrystalline Li–Al–O interphase. Subsequently, this ALD layer forms a multiphase microstructure during high-temperature sintering comprising LiAlO_2_, Li_2_ZrO_3_, and LaAlO_3_ with their phase fractions and spatial distribution being directly controlled by ALD coating thickness, enabling tunable densification and ion transport characteristics. Thickness-dependent regimes of sintering are introduced, which, evaluated by electrochemical experiments, show that medium-thickness coatings of ∼6.8 nm (25 ALD cycles) yield optimal performance. With a room temperature ionic conductivity of 0.39 mS cm^–1^ and a critical current density of 0.35 mA cm^–2^, they outperform both thinner and thicker coatings, as the former suffer from insufficient densification, while the latter suffer from phase overgrowth. This work provides mechanistic insight into the ALD-guided modification of the chemical and morphological landscape of garnet-type SSEs. More broadly, it establishes design principles for engineering interphases with tailored transport properties, offering a scalable and tunable strategy for advancing the performance of solid-state lithium metal batteries.

## Graphical abstract

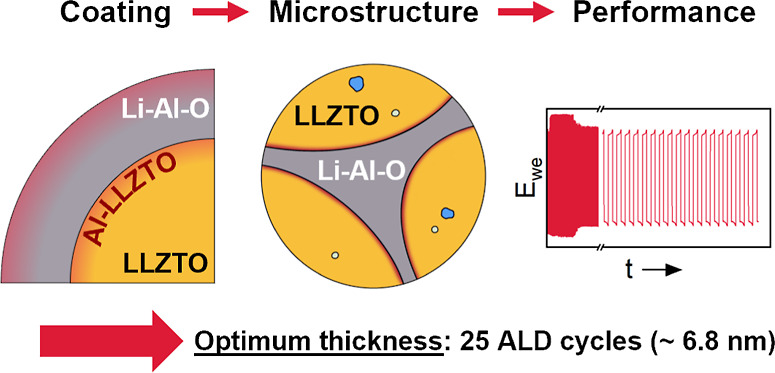

## 1 Introduction

The development of all-solid-state batteries has attracted significant attention as a promising next-generation energy storage technology due to their intrinsic safety, enhanced thermal stability, and potentially higher energy densities compared to conventional lithium-ion batteries.^1,2^ Among solid-state electrolyte (SSE) materials, garnet-type Li_7_La_3_Zr_2_O_12_ and its doped derivatives, e.g., Ta-doped Li_6.4_La_3_Zr_1.4_Ta_0.6_O_12_ (LLZTO), have emerged as leading candidates owing to their high ionic conductivity and mechanical strength, as well as their good chemical stability, especially against metallic lithium, and wide electrochemical stability window.^3–6^ Despite these advantages, the practical implementation of garnet-type SSEs remains limited by severe interfacial challenges. Rigid oxide electrolytes often form point contacts with electrodes, resulting in poor interfacial contact and elevated resistance. Additionally, surface contaminants, grain boundaries, and phase heterogeneity can further reduce interfacial stability and promote lithium dendrite formation, compromising the mechanical integrity and cycling stability of the electrolyte.^4,7–11^

Overcoming these challenges requires precise interface engineering strategies that can simultaneously improve densification, enhance ionic transport, and suppress the formation of parasitic phases and Li-dendrites. Commonly reported strategies try to mitigate these challenges through the utilization of different sinter additives, such as Al_2_O_3_, LiAlO_2_, CuO, or liquid metallic gallium.^12–15^ By means of physical mixing, these additives are introduced between the garnet-type powder particles, promoting liquid-assisted densification during high-temperature sintering. Furthermore, after sintering, garnet pellets can also be mechanically polished to remove surface contaminations and to improve contact between the electrodes and the SSE.^4,7^ However, both strategies tend to uncontrollably introduce large amounts of secondary phases or cracks, degrading long-term performance. Therefore, interface modification via 2D-interlayer or thin-film coating application on pellet-level has been exploited.^16–18^ While this concept provides more effective protection against lithium dendrite growth without parasitic interphase formation, it is still mostly limited to the LLZTO||electrode interface and lacks optimization of interfaces inside the garnet-type SSE, which are equally crucial for overcoming the previously mentioned challenges.

One effective approach for tailoring such internal interfaces is the application of thin-film coatings at powder-level.^19–21^ For example, wet-chemical coating processes are frequently employed due to their cost-effectiveness and suitability for large-scale production. However, solution-based methods exhibit limited control over film morphology and conformity. Consequently, nonuniform surface coatings can result in inhomogeneous distribution and, potentially, promote agglomeration during subsequent processing. Furthermore, battery materials often require only Å-level thickness coatings for significant performance improvements. Among the available coating techniques, only atomic layer deposition (ALD) offers the required process characteristics, such as conformal coverage over complex geometries and precise thickness control at subnanometer scale.^22–26^ Even though powder ALD requires significant precursor consumption compared to pellet-level ALD, its superior process control offers near-100% precursor usage, demonstrating significantly reduced precursor waste compared to wet-chemical processes.^25,27^ Moreover, recent advances in reactor design and process engineering have significantly improved scalability of powder ALD. Approaches, such as atmospheric-pressure ALD, fluidized-bed and rotary reactors, and spatial ALD systems, enable high-throughput production (kg to tons), making it highly relevant for powder-level surface modification.^28–31^

ALD has been successfully applied to LLZTO powder, where Al_2_O_3_ coatings act as a homogeneously distributed sinter additive, facilitating densification and reducing grain boundary resistance via a liquid-phase sintering process.^21^ However, this previous study has solely focused on ALD-enhanced densification, with limited exploration of its influence on interfacial chemistry, particularly during the coating process itself. Emerging studies suggest that ALD coatings on lithium-containing substrates can induce the formation of compositionally complex interphases. In cathode materials, for instance, Al_2_O_3_ coatings have been shown to react with surface carbonates and hydroxides to form Li–Al–O-rich interlayers.^32^ Furthermore, these interlayers can also be formed as a result of lithium diffusion caused by reactions (e.g., electrostatic interactions) between the substrate material and ALD precursors.^33,34^ In the context of garnet-type SSEs, such interactions may be further amplified by the high lithium mobility of the host structure, potentially giving rise to graded, lithium-rich interphases that evolve during high-temperature sintering into multiphase structures with significant influence on electrochemical behavior. Despite these indications, detailed mechanistic understanding of interphase evolution during ALD and sintering remains limited. Key questions persist regarding the extent of lithium diffusion during ALD, the crystallinity and phase identity of resulting interlayers, and how coating thickness modulates interfacial chemistry, phase transformation pathways, and functional properties such as ionic conductivity and dendrite suppression.

In this work, ALD was utilized to apply uniform Al_2_O_3_ coatings on LLZTO powder particles, creating a core–shell structure, which aims to elucidate the interfacial phenomena that occur during deposition and subsequent densification. The study investigates how lithium diffuses into the alumina layer during ALD, leading to the formation of a compositionally graded Li–Al–O interphase. Postsintering structural analyses reveal the emergence of multiphase microstructures, including LiAlO_2_, Li_2_ZrO_3_, and LaAlO_3_, with their concentrations strongly dependent on the initial coating thickness. Electrochemical testing emphasizes the impact of these interfacial and microstructural changes on ionic/electronic conductivity and critical current density (CCD). Finally, new thickness-dependent sintering regimes were introduced as guideline for ALD-based optimization of high-performance garnet-type SSEs.

## 2 Results and Discussion

### 2.1 Li–Al–O Interphase Formation on LLZTO after Powder ALD

The morphology and chemistry of the applied nanolayer, deposited through 100 ALD cycles, were investigated to understand its underlying growth mechanism. Therefore, high-resolution (HR) scanning transmission electron microscopy (STEM) and advanced spectroscopic methods, such as nuclear magnetic resonance (NMR) and X-ray photoelectron spectroscopy (XPS), were applied to analyze conformity, interfacial bonding, structural order, thickness, and chemical composition.

By HR bright field (BF) STEM imaging of an ALD-coated LLZTO particle (Figure 1a), an approximately 24.5 ± 0.8 nm thick layer with a sharp contrast to the low-intensity, heavy element-containing LLZTO particle can be observed. The nanolayer appears free of cracks or pinholes and exhibits a predominantly amorphous structure with slight evidence of ordering close to its edge, as confirmed by the diffraction spots obtained from fast Fourier transform (FFT) analysis of the HR-BF image from Figure 1a (see Figure 1b). The induced structural order is likely to be associated with the high number of applied ALD cycles and the stop-flow deposition mode, which results in an extended deposition duration of approximately 80 h. The corresponding input of thermal energy increases atomic mobility and promotes atomic rearrangement, leading to the nucleation of nanocrystalline domains within the amorphous coating. A detailed examination, including line profiles of the observed ordered regions, can be found in the Supporting Information (Figure S1). The lengths between the reciprocal lattice points |2 ${\vec{g}}_{hkl}$ | were measured and correspond to lattice fringes of *d*_*hkl*_ = 4.43 Å (FFT#1), *d*_*hkl*_ = 2.50 Å (FFT#2), and *d*_*hkl*_ = 3.03 Å (FFT#2), closely matching the values obtained from the frequencies of the line profiles in the real space image over 5 or 10 planes (Table S1). Table S2 lists the possible indexing of the derived lattice plane distances with standard α-Al_2_O_3_ and monoclinic θ-Al_2_O_3_, which were reported for crystallized alumina ALD coatings.^35^ However, it is evident that the observed lattice plane distances cannot be fully explained by either structure. A more discrete discrimination would only be possible with nanocrystalline regions close in zone axis orientation, which were not observed in this study.

**Figure 1:**
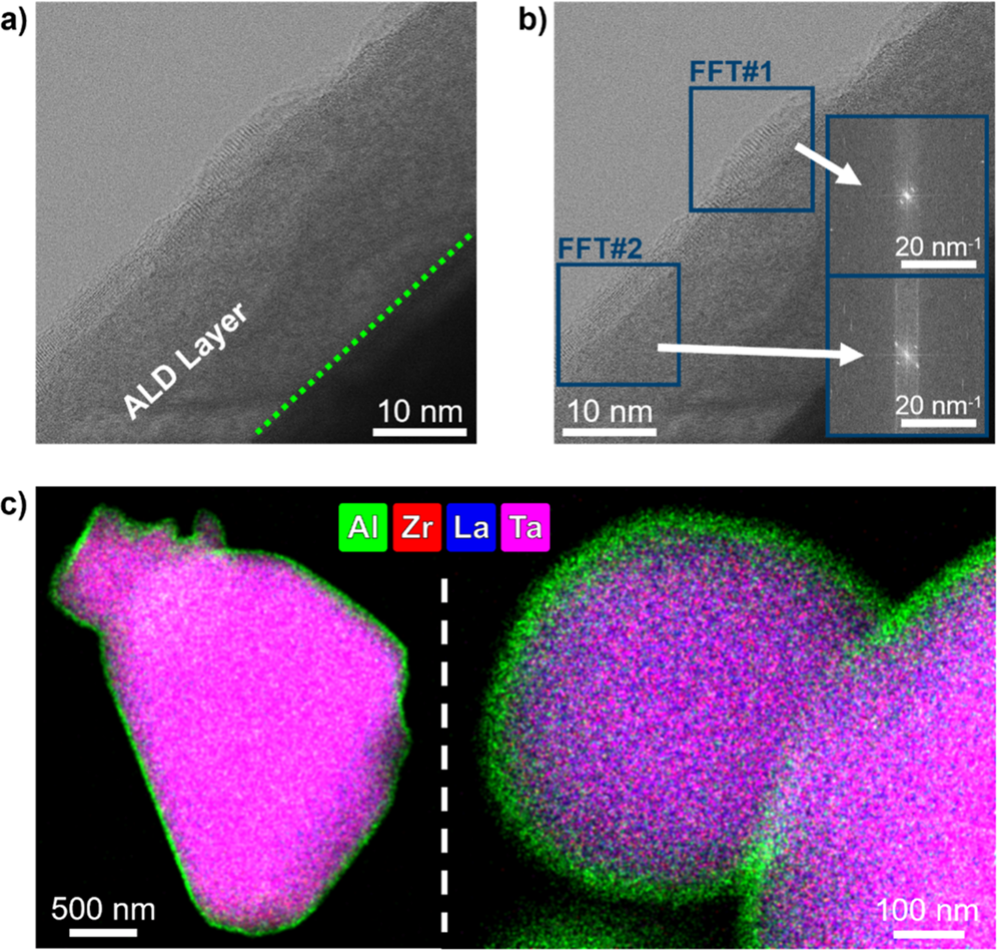
(a) HR-BF image of 100 ALD cycles coated on LLZTO powder particles; (b) FFT analysis on HR-BF STEM image in (a); (c) HR-EDS mappings at different magnifications (the O-signal was excluded to enhance clarity, given its presence in both the LLZTO particle and the ALD coating).

Energy-dispersive X-ray spectroscopy (EDS) mappings (Figure 1c) depict an Al-rich layer which is spatially confined along the surface of individual particles, representative of a core–shell structure. The aluminum signal sharply marks the coating boundary and resembles the clear contrast observed in the HR-BF image in Figure 1a, demonstrating that the deposition process effectively forms conformal and uniform nanolayers on the complex surface topology of LLZTO powder particles that are chemically well-defined and tightly bound to the LLZTO base material.

Based on the thickness of the ALD layer on the LLZTO particle surface, which was derived from HR-STEM imaging, the growth rate of the deposition process amounts to ∼2.5 Å/cycle. However, for reference, a planar Si wafer was simultaneously coated within the same deposition process and the resulting layer thickness of 15.86 ± 0.06 nm, as measured by spectroscopic ellipsometry (Supporting Information Figure S2), reveals a substantially reduced layer growth of ∼1.6 Å/cycle. Given that all samples were prepared under identical conditions, the origin of the different ALD growth rates can either be attributed to the different substrate geometries of the Si wafer and LLZTO powder or to differences in their chemistry. During the deposition onto powder particles, the surface area increases along with the process as the radius of a powder particle increases with increasing layer thickness. This can potentially lead to more available surface sites and, thus, more precursor adsorption, ultimately enhancing growth rates. However, the continuous coating process also promotes agglomeration, which actively reduces surface area and may partially offset the surface-enhanced growth factor. To examine the extent of this effect, 100 ALD cycles of Al_2_O_3_ were deposited on silicon powder, serving as a 3D substrate with a chemical composition equivalent to that of silicon wafers (Figure S3). The layer thickness derived from the HR-BF STEM image in Figure S3b results in a growth rate of ∼1.2 Å/cycle on Si powder, similar to that on the Si wafer, indicating that the enhanced growth rate of Al_2_O_3_ on LLZTO mainly arises from chemical interactions at the interface rather than surface area effects. One potential explanation for a chemistry-based increase in layer thickness could be the diffusion of lithium from LLZTO into the growing Al_2_O_3_ layer during the deposition process which may result in the formation of a lithiated Al–O matrix (Li–Al–O).^33,36,37^

To confirm the presence of a potential Li–Al–O interphase on the LLZTO surface after deposition, the chemistry of the ALD layer was subsequently analyzed using ^27^Al magic angle spinning (MAS) NMR and XPS measurements of the Al 2p signal (Figure 2). Due to the mild deposition conditions (*T*_dep_ = 225 °C), diffusion of Al^3+^ into the LLZTO substrate is expected to be limited.^38^ Consequently, the ^27^Al environment will be dominated by the contribution of the ALD layer, thereby providing information about its chemical nature. The NMR spectrum of the 100-cycle sample in Figure 2a exhibits dominant resonances near 73 ppm, corresponding to tetrahedrally coordinated Al (Al^IV^), with additional signals at 35 ppm and −2 ppm attributed to penta- (Al^V^) and octahedral (Al^VI^) sites, respectively.^39,40^ When comparing the spectra with in-house acquired reference data of LiAlO_2_ powder and amorphous Al_2_O_3_ ALD coatings on Si powder (Figure 2b), the ^27^Al environment of ALD-coated LLZTO samples does not resemble a layer of pure, amorphous Al_2_O_3_ but instead the features align more closely with the chemical environment of LiAlO_2_,^41^ confirming lithium incorporation into the alumina layer. At the same time, the temperature treatment at 225 °C throughout the ALD process is expected to induce a reaction between the coating and the substrate, likely leading to the formation of a Li_6.4–3*x*_La_3_Zr_1.4_Ta_0.6_Al_*x*_O_12_ solid solution limited to the subsurface layer of the LLZTO particle.^42,43^ The Al inside the garnet lattice would be characterized by a peak at approximately 68 ppm.^44^ However, this peak cannot be clearly distinguished from the spectrum as structural disorder, typical of amorphous or partially disordered ALD-grown layers,^45^ induce spectral asymmetry and broadening toward lower chemical shifts, effectively overlapping possible signals from Al inside the LLZTO lattice. Furthermore, the spectrum also reveals a narrower Al peak overlapping with the broad resonance from the Al^IV^ site (Figure 2a), indicating the presence of nanocrystalline domains embedded within an otherwise amorphous matrix. This is consistent with the previously reported observation of partially ordered LiAlO_2_ phases exhibiting similar spectral features^41^ and confirms our finding of locally ordered regions, as revealed by FFT analysis and the line profiles in Figure S1. However, the three measured lattice distances do not fully correspond to those expected for α-LiAlO_2_. A comparison with other lithium aluminates, such as α-LiAl_5_O_8_ and α-Li_5_AlO_4_, also remains inconclusive as these structures share similar lattice plane distances (see Table S2). This suggests the presence of a more complex local structure throughout the ALD layer, which is why complementary XPS measurements (Figure 2c) were conducted to provide further chemical insight into the ALD-derived interphase. In accordance with previous studies,^46,47^ the Al 2p peak position obtained for ALD-coated LLZTO shifts significantly from the binding energy of pure ALD-grown Al_2_O_3_ on Si powder (∼74.4 eV) toward the one obtained for a LiAlO_2_ reference (∼73.8 eV). The peak is eventually centered at about 73.9 eV which clearly points to the formation of Li–Al–O bonds within the coating, in agreement with the NMR results. Furthermore, analysis of the C 1s spectrum of pristine LLZTO reveals the presence of residual Li_2_CO_3_ on the particle surface prior to deposition (see Figure S4). The carbonate species accounts for only 3.70% of the total C 1s signal, thereby making a negligible contribution to the overall surface composition. As can be seen in the HR-STEM–EDS data (Figure 1), the ALD coating appears conformal and uniform on the LLZTO particle, indicating that minor residual Li_2_CO_3_ does not exert a detrimental effect on layer growth. These findings suggest, in agreement with previous reports, that exposure to trimethylaluminum during ALD can effectively reduce Li_2_CO_3_, thereby exposing fresh LLZTO surfaces for subsequent layer formation.^33^

**Figure 2:**
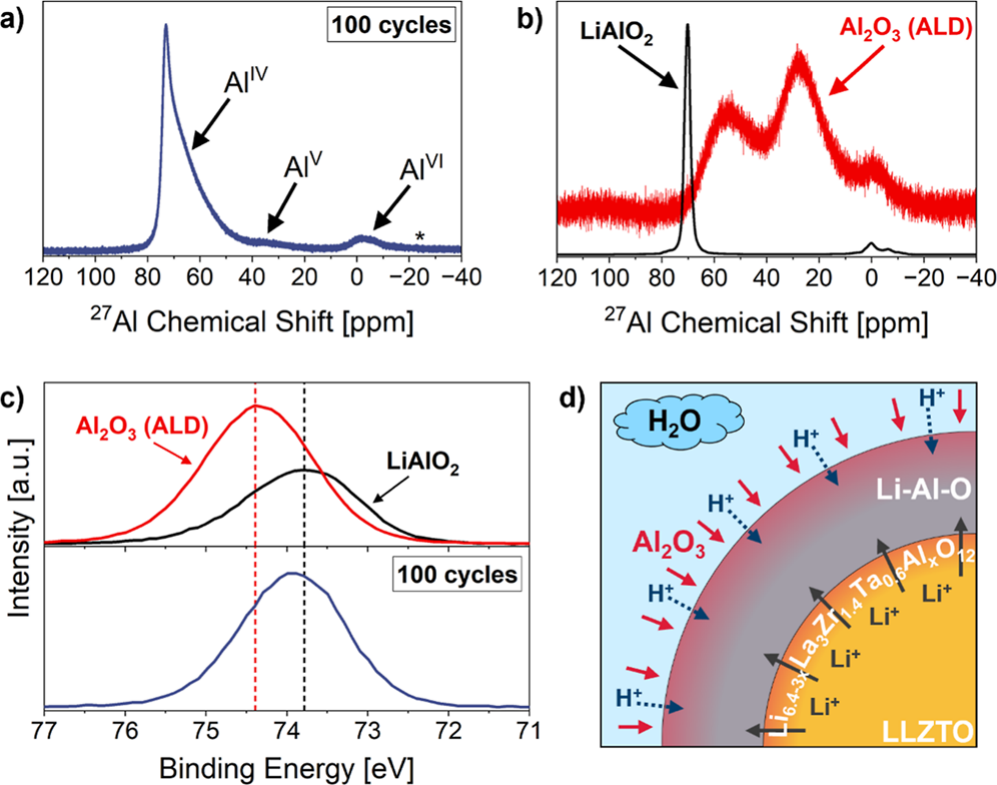
(a) ^27^Al MAS NMR spectrum of ALD-coated LLZTO powder (100 cycles) (asterisk: spinning sideband); (b) normalized reference spectra measured using LiAlO_2_ powder (black) and Al_2_O_3_ ALD-coated (100 cycles) Si powder (red, vertically shifted for clarity); (c) XPS HR-scan of the Al 2p regions of ALD-coated LLZTO powder (100 cycles) (bottom graph, blue), LiAlO_2_ powder (top graph, black), and Al_2_O_3_ ALD-coated (100 cycles) Si powder (top graph, red); (d) schematic illustration of the expected layer formation of compositionally complex Li–Al–O coatings on LLZTO powder.

While literature reports have identified Li–Al–O interfacial layers with typical thicknesses of ∼2 nm on Li-based cathodes during Al_2_O_3_ ALD,^32–34^ the evidence here, based on the 100 ALD cycles reaching 24.5 nm yet still exhibiting strong lithiation signals (Figure 2), points to a much more profound and extensive lithiation zone. This implies the presence of an additional driving force beyond electrostatic attraction or parasitic side reactions. Enhanced lithium diffusion from the bulk into such dense and “thick” Al_2_O_3_ layers might be attributed to the proton-lithium exchange mechanism reported for highly Li^+^ conducting LLZTO.^48–50^ Impurity H^+^ within the growing Al_2_O_3_ layer, as well as the gaseous H_2_O-atmosphere utilized as coreactant in the ALD process, have the potential to induce H^+^/Li^+^ exchange reactions at the LLZTO surface.^51^ This exchange creates a local lithium chemical potential gradient, causing Li^+^ to be pulled outward into the coating and forming a compositional gradient inside the Al_2_O_3_ layer itself. The lithium mobility across the interface between the ALD layer and LLZTO is further facilitated by efficient migration pathways through the Li_6.4–3*x*_La_3_Zr_1.4_Ta_0.6_Al_*x*_O_12_ solid solution, which is expected to form in the subsurface layer of LLZTO as a result of the applied deposition temperature of 225 °C.^42,43^ As the coating thickens, the potential gradient runs out, resulting in a top surface layer richer in Al and O, while the inner layer remains lithiated, as schematically illustrated in Figure 2d.

Overall, the discussed microscopic and spectroscopic results provide compelling, multimodal evidence that ALD of Al_2_O_3_ on LLZTO powder does not simply deposit a passive overlayer but rather induces active lithium migration, interphase formation, and local crystallization within the coating, resulting in a compositionally graded, partially nanocrystalline Li–Al–O layer. Such lithium-rich coatings may be more reactive or sinter-active, further influencing sintering behavior, phase evolution, and electrochemical performance, highlighting the central role of ALD as a design tool for solid-state battery interfaces.

### 2.2 Structural and Morphological Evolution of ALD-Modified LLZTO after Sintering

After the chemistry and morphology of the ALD layer on LLZTO powder has been discussed, its influence on the high-temperature sintering process (*T* = 1250 °C) needs to be investigated in order to introduce a guideline for ALD-assisted interface modification in garnet-type SSEs. Therefore, varying layer thicknesses were implemented by applying 10, 25, 50, and 100 ALD cycles, corresponding to 3.8 ± 0.2 nm, 6.8 ± 0.1 nm, 12.9 ± 0.4 nm, and 24.5 ± 0.8 nm, respectively, as obtained from HR-BF STEM images (Figures 1 and S5). X-ray diffraction (XRD) patterns of sintered LLZTO powders demonstrate that pristine samples maintain a predominant cubic garnet structure, while samples coated via atomic layer deposition exhibit the emergence of secondary phases (LiAlO_2_, Li_2_ZrO_3_, LaAlO_3_) whose abundance depends on the number of applied ALD cycles, creating multiphase structures (Figure 3). Quantitative phase analysis reveals that the total amount of secondary phases increases from 0.4 wt % in pristine LLZTO to 8.6 wt % in the 100-cycle sample. This progressive increase reflects the enhanced interfacial reactivity between the LLZTO surface and the Li–Al–O containing ALD coating under the given sintering conditions.

**Figure 3:**
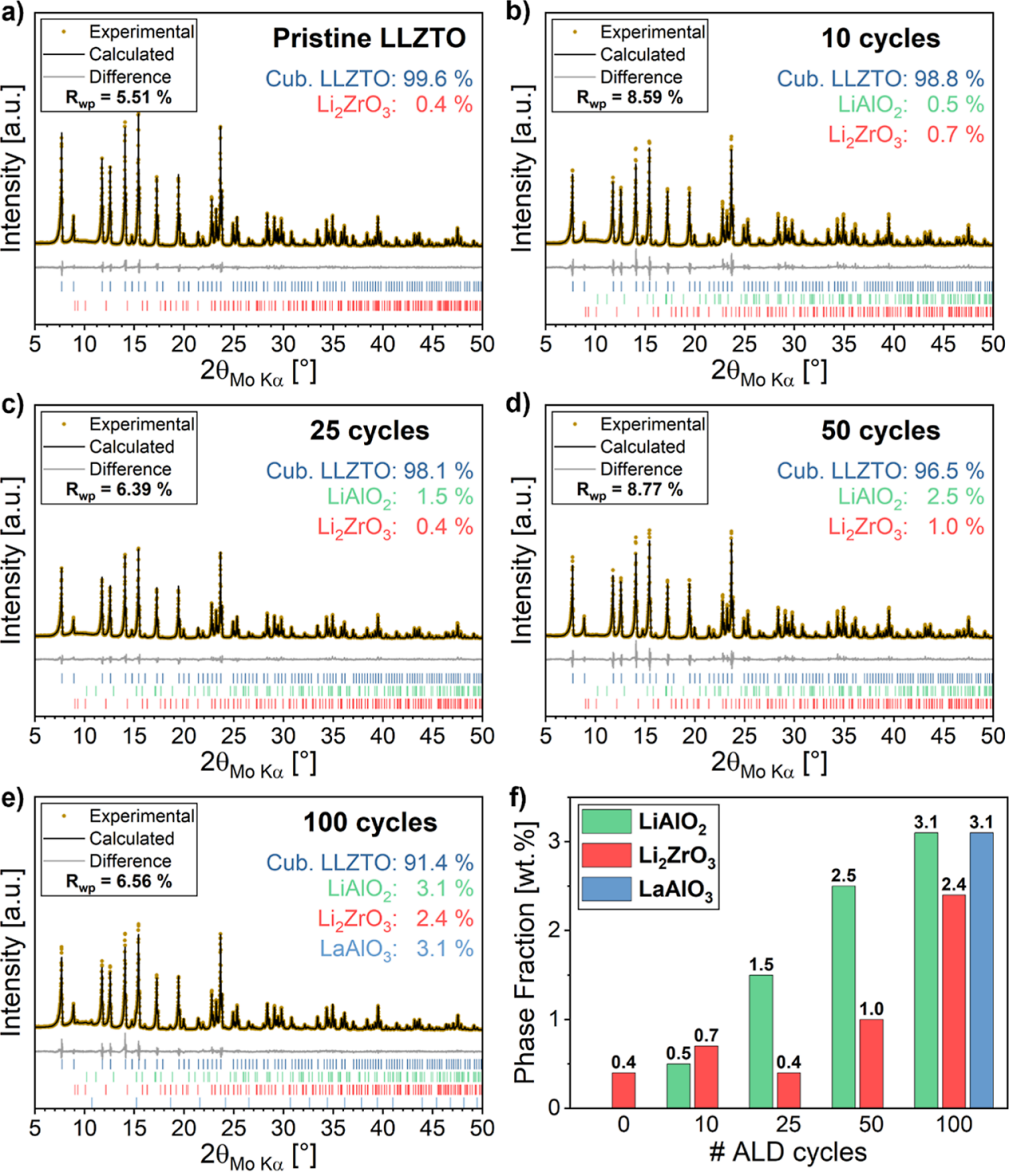
Quantitative XRD phase analyses of pristine (a), 10-cycle (b), 25-cycle (c), 50-cycle (d), and 100-cycle (e) samples after sintering at 1250 °C; (f) overview of determined secondary phase fractions with respect to the amount of applied ALD deposition cycles.

As illustrated in Figure 3f, Li_2_ZrO_3_ (ICSD# 291154) can be consistently observed across all diffractograms and is attributed to the partial decomposition of the LLZTO matrix at the elevated sintering temperature of 1250 °C, similar to previous reports highlighting Zr-containing decomposition pathways in LLZTO.^52–55^ LiAlO_2_ (ICSD# 23815), however, is detected only in ALD-coated samples, which results from Li^+^/Al^3+^ interdiffusion between the Li–Al–O layer and the LLZTO structure. This reaction closely resembles the formation of a Li_2_O–Al_2_O_3_ eutectic, as extensively reported for Al_2_O_3_ sinter additives introduced via physical mixing into LLZTO.^46,56–58^ For material coated with 100 ALD cycles, approximately 3.1 wt % LaAlO_3_ (ICSD# 170772) appear in addition to Li-containing secondary phases, suggesting that excess Al results in increased interdiffusion into the LLZTO lattice, exceeding its solubility limit and, thus, promoting the formation of the perovskite-type phase.^59–61^ Moreover, in these samples a substantial increase in Li_2_ZrO_3_ phase fraction to 2.4 wt % can be observed as the formation of LaAlO_3_ is associated with coordination rearrangements of La atoms and, therefore enhanced ZrO_6_ octahedral distortion, effectively destabilizing the garnet matrix.^62^ The absence of La_2_Zr_2_O_7_, a phase typically linked to bulk Li loss, suggests that lithium depletion remains localized at grain boundaries and that the bulk garnet framework is preserved.

In addition to XRD phase analysis, the influence of the ALD coatings and their varying thickness on the microstructural evolution of sintered LLZTO pellets was investigated by SEM–EDS measurements (Figures 4 and S6). Compared to pristine LLZTO, samples coated with 10, 25, and 50 ALD cycles exhibit enhanced sinter activity, reaching high levels of densification (relative densities >90%), peaking at 95.05 ± 0.30% for the 50-cycle sample (Figure S6a). In SEM they display well-developed microstructures via homogeneous grain growth with grain sizes well above 50 μm, attributable to surface and lattice diffusion as induced by the elevated sintering temperature of 1250 °C. Apart from surface defects introduced by the polishing process required for sample preparation, the micrographs display no obvious defects, e.g., microcracks, originating from internal stresses that could accumulate during high-temperature sintering. The reason for that could be the homogeneous distribution of the coating, as facilitated by the powder-level ALD process, which is reported to be detrimental to reducing residual stress.^21,63^

**Figure 4:**
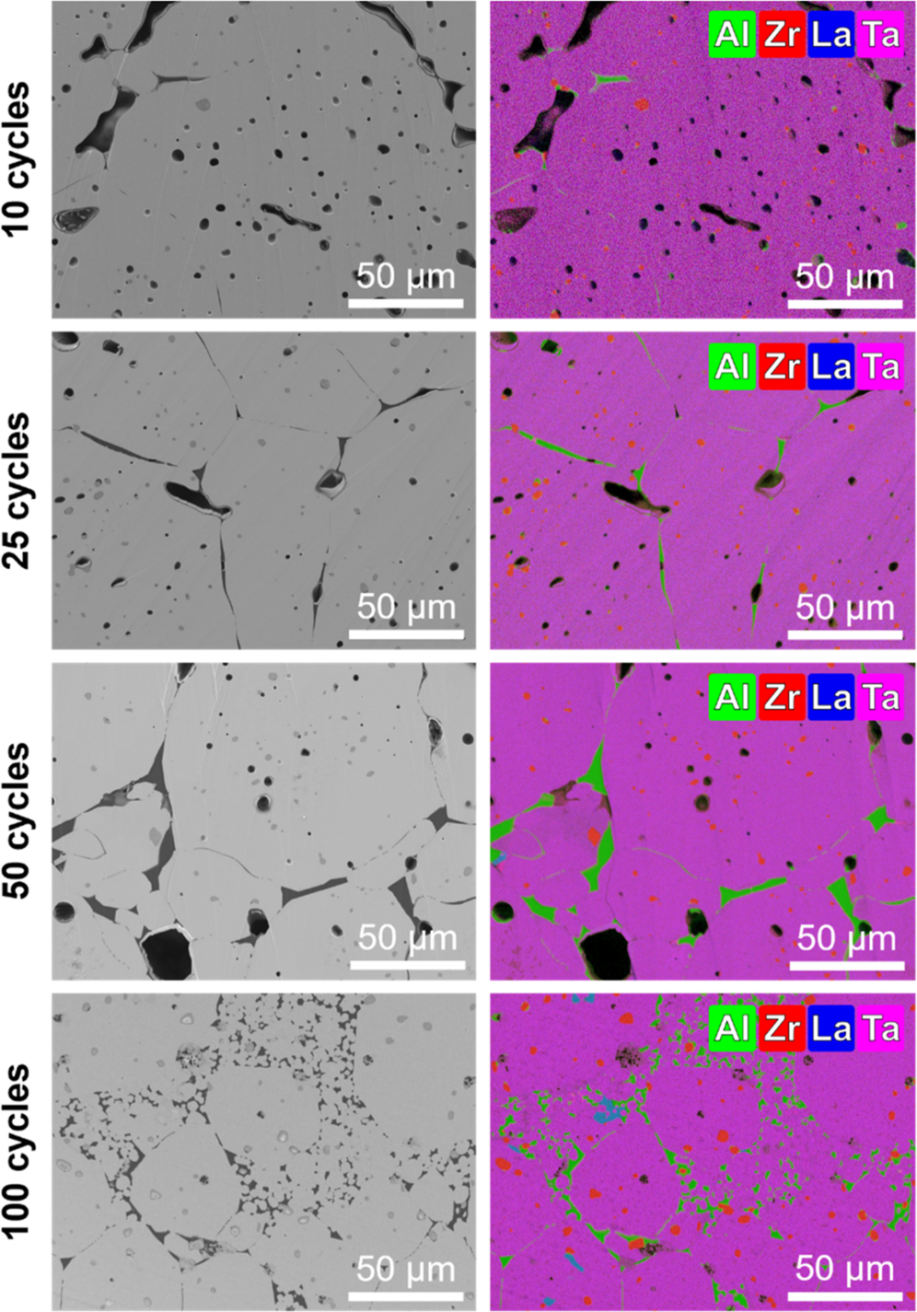
SEM and EDS results obtained from LLZTO pellets after sintering of ALD-coated powders with different ALD cycle numbers (the O-signal was excluded to enhance clarity, given its presence in all phases.).

As the number of applied ALD cycles increases, pores, triple junctions, and grain interfaces are increasingly occupied by a dark interphase, which is shown by EDS mappings to be Al–O-rich. Its phase composition (Al/O-ratio: ∼1:2) corresponds to the thermodynamically stable LiAlO_2_ phase observed in the XRD data (Figure 3) and results from Li^+^/Al^3+^ interdiffusion between the Li–Al–O coating and the LLZTO host structure. The ALD layer acts as sintering additive by transforming into a liquid-phase Li_2_O–Al_2_O_3_ eutectic that introduces capillary forces, promoting densification and the formation of large, well-connected grains. Consequently, the identification of heterogeneous grain interfaces is becoming increasingly evident in relation to coating thickness. However, for the highest number of applied ALD cycles (100), a different behavior is observed. Although the coating still promotes initial densification, the microstructure becomes heterogeneous after sintering. The grain size distribution broadens significantly, with microsized grains located at grain boundaries of larger ones. EDS analysis reveals that accumulated secondary phases, particularly LiAlO_2_ and LaAlO_3_, segregate along grain interfaces. As LaAlO_3_ has a high melting point of ∼2080 °C, the solid phase could hinder grain coalescence and facilitates the formation of isolated grains and pores, as reflected in a minor decline in relative density to ∼93.5%.^56^ Moreover, all EDS mappings show Zr–O-rich precipitations inside LLZTO grains, in accordance with the detected high-temperature decomposition phase Li_2_ZrO_3_.

In contrast, the microstructure of pristine LLZTO (Figure S6b), exhibits poor and inhomogeneous densification with substantial residual porosity, reflected in a low relative density of ∼75% (Figure S6a). Because the material is insufficiently sintered and, therefore, relatively soft, the microstructure is not as clearly visible as in the ALD-coated samples. However, a smaller grain size can be observed and in addition to that, elemental mappings of the O- and C-signal (Figure S6c,d) give evidence of Li_2_CO_3_ in open pores. Since surface carbonates cannot be removed in pristine LLZTO without the addition of sinter additives as the required decomposition temperature lies at ∼1600 °C, the carbonate-species melts during sintering (*T*_m_ = 723 °C), covers the surface of adjacent LLZTO particles and separates them, effectively impeding mass transport.^56^ Thus, without modification using ALD, sintering of the pristine material is limited to surface diffusion, resulting in reduced grain growth and insufficient densification.^63^

The extent of Li^+^/Al^3+^ interdiffusion between the Li–Al–O coating and LLZTO during sintering was exemplarily investigated through HR-STEM–EDS analysis on a lamella extracted from the 25-cycle sample (Figure 5). In the first step, low-magnification measurements were conducted to examine Al-diffusion into the LLZTO grain on the micrometer scale. The BF-image and the EDS data reveal a distinct contrast between the Al-rich grain interphase, corresponding to LiAlO_2_, and the garnet phase. The derived line scan shows that the Al-signal sharply drops at the Li–Al–O/LLZTO interface without significant decay over the entire investigated range of ∼3.2 μm inside the grain. The signal of aluminum inside bulk LLZTO could originate from fluorescence from Al-containing parts of the STEM-holder, as similar concentration levels were also observed in other experiments using this setup on samples without aluminum. Furthermore, there is no clear indication that Al has diffused from the intergranular phase into the bulk as the Al-signal does not resemble a well-defined, long-range diffusion gradient. Subsequently, nanometer-scale measurements were performed to further assess local interdiffusion at the Li–Al–O/LLZTO interface (see Figure 5b). The EDS line profile demonstrates that, in accordance with the intensity signal from the BF-image, the Al-concentration rapidly declines over a short-range of only 20 nm. In combination with the relatively short dwell time of 75 min during high-temperature sintering, the STEM–EDS results indicate that Li^+^/Al^3+^ interdiffusion is locally constrained to the vicinity of the Li–Al–O coating rather than introducing notable bulk Al-doping in LLZTO.

**Figure 5:**
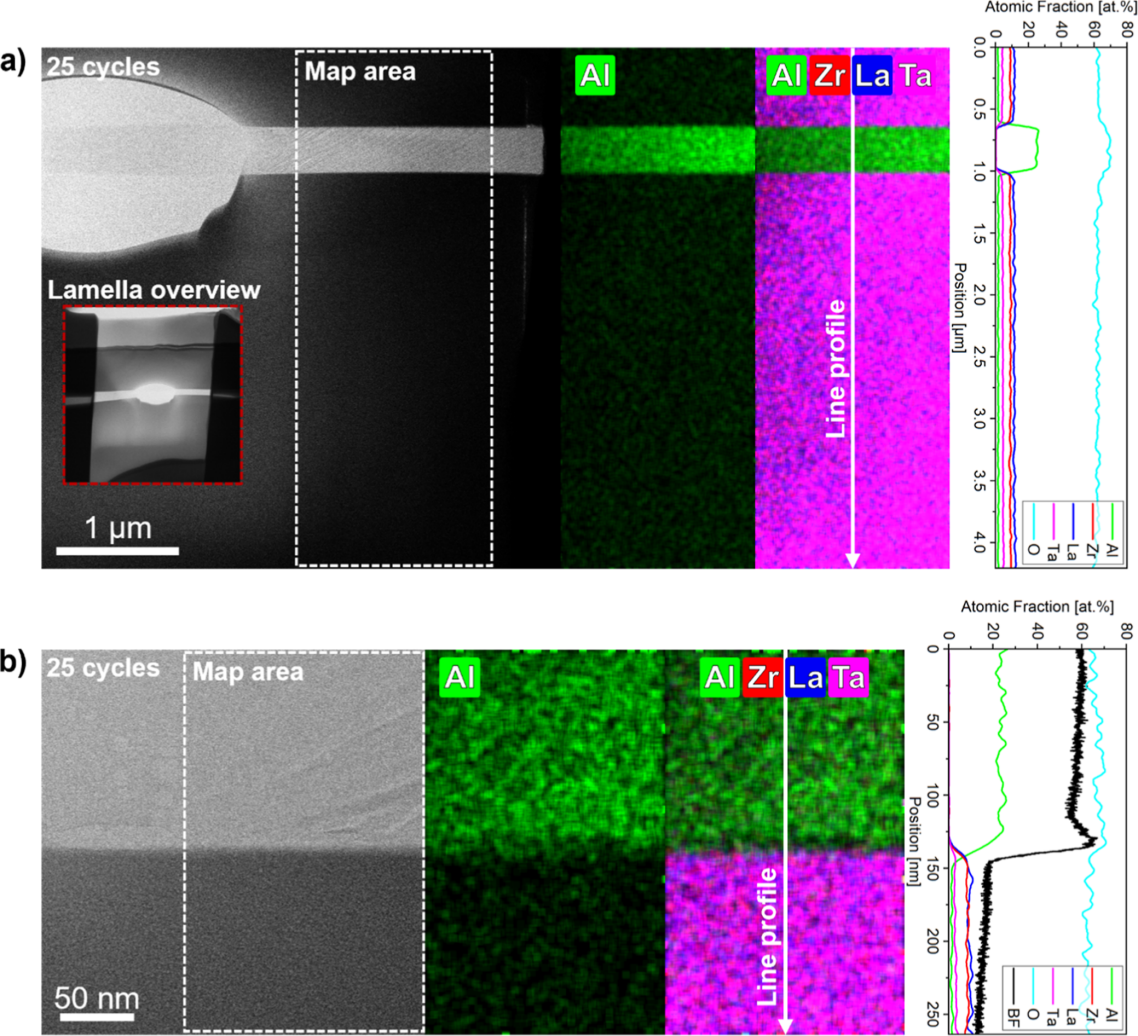
HR-STEM–EDS analysis on Li^+^/Al^3+^ interdiffusion on a lamella extracted from sintered LLZTO (25 cycles); (a) investigations in μm-range; (b) investigations in nm-range (the O-signal was excluded from mappings to enhance clarity, given its presence in all phases.).

To further elucidate the mechanistic origins of the observed structural evolution, grain growth behavior, and microstructural inhomogeneity during sintering, we propose three thickness-dependent sintering regimes for ALD-coated LLZTO (Figure 6): thin coatings (<25 ALD cycles), medium-thickness coatings (25–50 ALD cycles), and thick coatings (>50 ALD cycles). Each regime exhibits distinct interfacial behavior during thermal treatment, controlled by Li^+^/Al^3+^ interdiffusion, interfacial reactions, and grain boundary dynamics.

**Figure 6:**
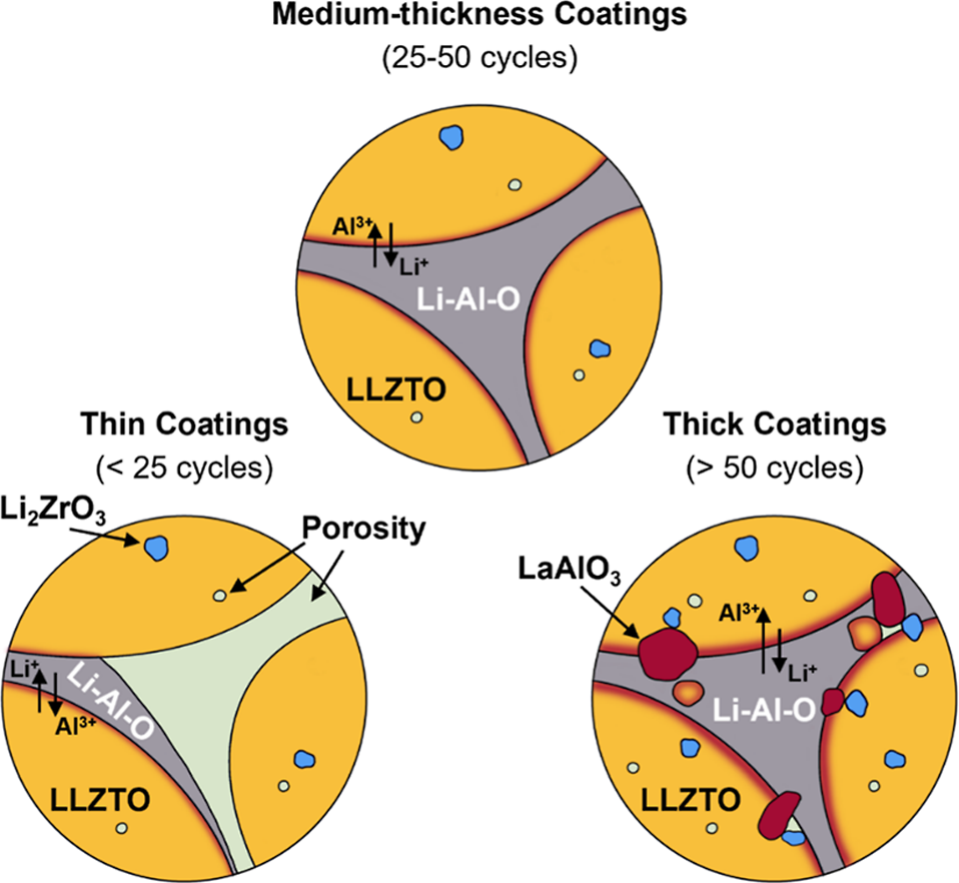
Schematic illustration of the proposed thickness-dependent sintering regimes in ALD-coated LLZTO.

Through the application of thin coatings (<25 ALD cycles), densification is improved with respect to pristine LLZTO. This can be attributed to the formation of a liquid and predominantly amorphous Li–Al–O interphase (Li_2_O–Al_2_O_3_ eutectic, *T*_m_ = 1055 °C) stemming from the ALD coating and local Li^+^/Al^3+^ interdiffusion with LLZTO, which induces liquid-phase sintering, facilitating particle rearrangement and enhancing mass transport via capillary forces and grain boundary diffusion, respectively.^46,60^ This reduces the surface free energy and promotes neck formation between adjacent grains as well as coalescence. According to Herring’s scaling law for sintering^64^

1 $$\frac{dR}{dt}\propto \frac{\gamma \Omega }{kT}\cdot \frac{D}{R^{2}}$$

where *R* is the particle radius, γ is surface energy, Ω is atomic volume, *D* is the effective diffusion coefficient, *k* is the Boltzmann constant, and *T* is temperature, the addition of liquid Li–Al–O locally enhances *D*, particularly via grain boundary diffusion, consequently accelerating densification and grain growth. As a result, the system undergoes uniform grain coarsening while maintaining structural coherence. However, samples in this regime still contain considerable volume fractions of porosity. During grain growth the limited amount of liquid Li–Al–O can no longer cover the entire grain surface area, which, besides liquid-phase sintering, additionally introduces solid-state sintering characteristics, causing the formation of a mixture of homogeneous and heterogeneous grain interfaces. Thus, the thin coating regime is governed by a mixture of solid-state and liquid-phase sintering.

In the medium-thickness regime (25–50 ALD cycles), grains grow extensively (>50 μm) with minimal porosity and excellent densification (∼95%), while being predominantly separated by heterogeneous grain interfaces (Figure 4). Therefore, the Li–Al–O ALD layer reaches an optimum thickness between 25 and 50 cycles, facilitating robust interfacial contact, while simultaneously maintaining thermodynamic phase stability. In this context, the Li–Al–O interphase acts as a diffusion bridge between grains without overloading the surface-near region of LLZTO with excess aluminum. Medium-thickness coatings establish a regime of interfacial equilibrium and optimal mass transport, in which liquid-phase-assisted sintering prevails as the dominant mechanism.

Thick coatings (>50 ALD cycles) of Li–Al–O form vast amounts of liquid phase. Interdiffusion from the extensive Al reservoir causes the solubility limit in the subsurface of LLZTO to be exceeded, resulting in the observed crystallization of LaAlO_3_.^59,60^ Its phase formation disrupts the thermodynamic equilibrium of the garnet lattice, increasingly triggering the formation of intragranular secondary phases (i.e., Li_2_ZrO_3_). The presence of solid LaAlO_3_ (*T*_m_ = 2080 °C) impedes grain coalescence and causes asymmetric interdiffusion between Al^3+^ and Li^+^ which, according to the Kirkendall effect,^65^ introduces phase boundary pinning and, subsequently porosity. Besides the influence on the thermodynamic equilibrium of the system, excess liquid phase during sintering also shows detrimental kinetic effects. First, grains can be isolated by the Li–Al–O melt, thereby reducing grain boundary area and mass transport. Second, grain boundary mobility is highly dependent on the amount of liquid. Thus, with thicker coatings, grain growth and densification occur too rapidly, leading to the entrapment of pores within the material.^57,60^ As a result, although densification remains relatively high (∼93.5%), the internal grain connectivity deteriorates. Hence, when the optimal ALD thickness is exceeded, dramatic shifts in thermodynamic and kinetic behavior occur, which is why the thick coating regime is characterized by oversaturation and multiphase formation.

### 2.3 Electrochemical Performance of ALD-Modified LLZTO Solid-State Electrolytes

The detailed structural and morphological characterization clearly reveals how modifications to the LLZTO surface induced by the atomic layer deposition process dramatically influence sintering behavior, phase formation, and grain connectivity. The formation of secondary phases, such as LiAlO_2_, Li_2_ZrO_3_, and LaAlO_3_, at varying ALD coating thicknesses, along with their spatial distribution at grain boundaries and interfaces, directly impacts not only the mechanical integrity of the sintered pellets but also the continuity of lithium and electron conduction pathways. These structural changes, including varying degrees of densification and intergranular phase segregation, are expected to play a decisive role in the electrochemical performance of the material. In particular, factors, such as interface resistance, electronic insulation by secondary phases, and interface adhesion strength between grains and interphases have the potential to exert a significant impact on lithium dendrite tolerance.^21,66^ To investigate these effects and evaluate the implemented thickness-dependent sintering regimes, the electrochemical performance of the sintered LLZTO samples was assessed by electrochemical impedance spectroscopy (EIS) and chronoamperometry (CA) measurements, as well as CCD and long-term cycling tests (Figure 7).

**Figure 7:**
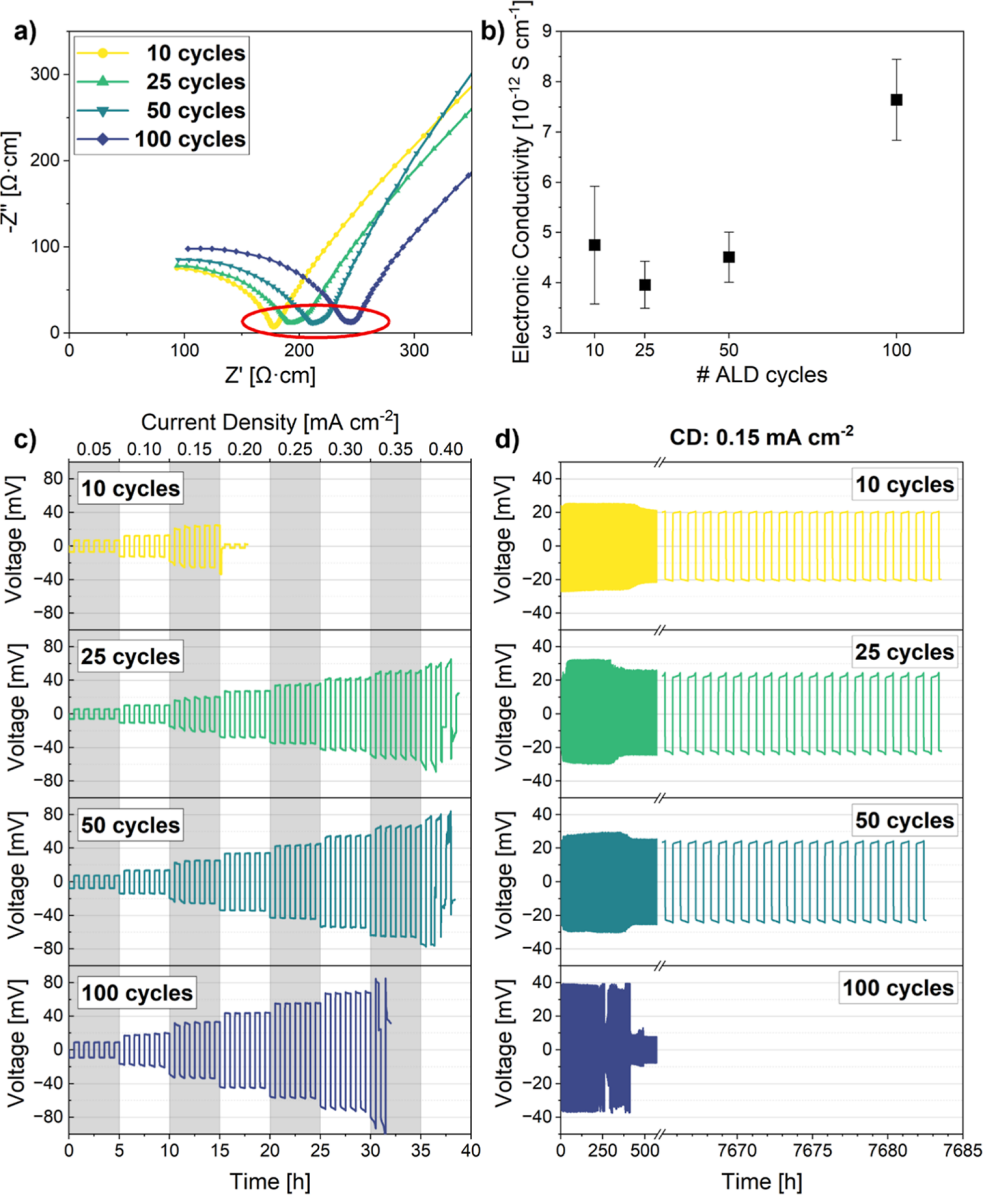
(a) Nyquist plots obtained from EIS measurements at 25 °C; (b) electronic conductivities derived from CA measurements at 25 °C; voltage profiles of Li||LLZTO||Li symmetrical cells for (c) CCD tests (step size: 0.05 mA cm^–2^ every 5 plating/stripping cycles, starting at 0.05 mA cm^–2^) and (d) long-term plating-striping experiments (CD: 0.15 mA cm^–2^).

Figure 7a depicts Nyquist plots obtained from room temperature EIS measurements on LLZTO pellets fabricated from ALD-coated powders using Au blocking electrodes. A partial semicircle in the high-frequency region is evident in all samples followed by a tail toward low frequencies, attributable to the contributions of the LLZTO sample and blocked Li-ions at the LLZTO/Au interface, respectively. Impedances of LLZTO grains and their interfaces/grain boundaries could not be clearly distinguished as separate semicircles, which is why the obtained data were only fitted for the total conductivity of the material correlated to the *x* intercept of the semicircle in the red-highlighted region.^66^ As the number of ALD coating cycles increases, the total conductivity at room temperature progressively decreases from 0.41 mS cm^–1^ for 10 cycles to 0.39 mS cm^–1^, 0.37 mS cm^–1^, and 0.33 mS cm^–1^ for 25, 50, and 100 cycles, respectively. These values are comparable to those reported in the literature for ALD-modified LLZTO (0.40–0.70 mS cm^–1^), considering slight differences in the respective sinter process (temperature, dwell time).^21,66^ Although porosity reduces from the thin (9.5%) to medium-thickness (4.9%) coating regime—a trend that would typically be expected to enhance Li^+^ mobility^21^—a reduction in the conductivity is observed, suggesting that mechanical integrity, e.g., porosity, plays a minor role in the measured samples. Furthermore, the substantial formation of secondary phases, such as Li_2_ZrO_3_ and LaAlO_3_, in the thick coating regime (>50 ALD cycles), is not reflected in the EIS data either, which display an almost linear decline in conductivity without any abrupt drop from 50- to 100-cycle samples.^60^ Lastly, Li^+^/Al^3+^ interdiffusion between the ALD coating and LLZTO could induce a positive impact on ionic conductivity through Al-doping.^59,60^ In case of relevant bulk modification of LLZTO with Al, a significant improvement in conductivity is expected. However, this expectation is in clear contrast to the experimentally observed overall linear decrease of ∼20%, thereby supporting the interpretation from the HR-STEM–EDS analysis in Figure 5, in which interdiffusion is observed to be confined to the subsurface of LLZTO grains, thus not exerting a notable influence on the electrochemical performance of the material. These findings indicate that the dominating factor for Li^+^ conduction in ALD-modified LLZTO is the presence of LiAlO_2_ at LLZTO grain interfaces, acting as physical barrier.^12^ This is also reflected in the microstructural analysis presented in Figure 4. In the thin coating regime, the amount of LiAlO_2_ is not sufficient to cover all interfaces between LLZTO grains, resulting in a microstructure consisting of a mixture of heterogeneous and homogeneous grain interfaces. The latter presents a lower barrier for Li^+^ transport, which is why 10-cycle samples were found to yield the highest observed conductivity. As the number of ALD coating cycles increases, both the quantity and thickness of heterogeneous interfaces grow, progressively impeding Li^+^ conduction. The increasing influence of interfaces is also apparent in the Nyquist plots, where deformations in the red-highlighted medium-frequency region become more pronounced. Additionally, the corresponding Arrhenius plots, derived from EIS measurements conducted over a temperature range of −100 to +100 °C and shown in Figure S7, reveal consistent activation energies of 0.43 ± 0.01 eV, indicating no significant variations and thereby confirming that bulk conduction through LLZTO remains energetically dominant. This lends further support to the conclusion that the observed changes in ionic conductivity with respect to ALD coating thickness refer to Maier’s brick-layer model,^67^ according to which the LiAlO_2_-interphase impedes Li-ion mobility, thereby increasing interface resistance. However, due to its substantially lower ionic conductivity and lack of temperature-dependent behavior, its effect is not captured in the activation energy trends.

In addition to that, the electronic conductivity of ALD-modified LLZTO was assessed by chronoamperometry testing (see Figure 7b). While the incorporation of insulating LiAlO_2_ has been reported to reduce electronic conductivity in LLZTO with increasing volume fraction,^21,66^ a nonmonotonic behavior can be seen in the present samples. As the number of ALD cycles increases from 10 to 25 cycles, the conductivity of the samples decreases from 4.75 × 10^–12^ S cm^–1^ to 3.96 × 10^–12^ S cm^–1^. However, beyond 25 cycles, an increase can be observed, culminating in a substantial improvement to 7.64 × 10^–12^ S cm^–1^ for 100-cycle samples. This phenomenon can be correlated with the microstructural analysis in (Figure 4). For instance, samples coated with only 10 ALD cycles exhibit pronounced variation, attributable to the relatively high porosity inside the material, which can influence the measurement through insufficient physical contact or local leakage currents. As the relative density increases, these effects are minimized, and a reduction can be observed in the presence of additional LiAlO_2_. However, the insulating effect is not as pronounced as expected due to enhanced grain connectivity. As the amount of LiAlO_2_ between LLZTO grains is further increased (50 cycles), it can be seen in the SEM micrographs in Figure 4 that percolating networks of these heterogeneous grain interfaces begin to form, providing continuous electron pathways and, thus, slightly increasing the conductivity again. In the 100-cycle sample, the percolating networks are found to be well-developed, which is why a substantial increase in electronic conductivity can be measured.^20^

The subsequent cell performance tests (CCD, long-term stability) demonstrate complex interactions between mechanical integrity and phase composition in ALD-coated LLZTO (Figure 7c,d). Samples from the thin coating regime (10 cycles), which exhibit the highest total ionic conductivity, were found to have the lowest tolerance against Li-dendrite growth (CCD: 0.15 mA cm^–2^). This can be explained by the relatively high porosity of the sample, which causes high tortuosity and uneven Li^+^ flow at the LLZTO/Li interface, as well as inside the LLZTO pellet. For 25- and 50-cycle samples, CCD notably improves to 0.35 mA cm^–2^ even though ionic conductivity is reduced. On the one hand, this can be attributed to the beneficial structural changes induced by enhanced sinter activity. As coating thickness increases, densification improves, leading to optimized mechanical stability, particularly in terms of adhesion strength at grain interfaces. On the other hand, the reduction of electronic conductivity is reported to impede the migration of free electrons, effectively reducing the probability for nucleation of metallic lithium along grain interfaces.^20,21^ Thus, both enhanced mechanical strength and reduced electronic conductivity at predominantly heterogeneous grain interfaces in medium-thickness samples (25–50 ALD cycles) make the material less prone against Li-dendrite propagation. Consequently, at 0.15 mA cm^–2^, samples with medium-thickness and also thin coatings exhibit stable cycling for over 7500 cycles, with a minimum overpotential of ∼20–25 mV. A prolonged formation period of 400–500 plating/stripping cycles can be observed, which could be traced back to the mild cycling conditions (0.15 mA cm^–2^, 30 min per half-cycle). Although the LLZTO pellets were polished and coated with Au, surface contaminants, e.g., Li_2_CO_3_, and the rigid nature of the ceramic cause high interfacial resistance and nonuniform Li deposition. Over cycling, Li creep gradually improves the inhomogeneous contact between LLZTO and metallic Li, but due to the low areal capacity per half-cycle a significant number of cycles is required to establish a stable interface. In pellets fabricated from LLZTO powders coated with 100 ALD cycles, the CCD value slightly decreases to 0.30 mA cm^–2^, whereas in long-term experiments cell failure already occurs after approximately 250 plating/stripping cycles. This demonstrates the crucial drawbacks of the described multiphase structure, stemming from excessive interdiffusion and interfacial reactions in the regime of thick coatings. The formation of LaAlO_3_ and Kirkendall porosity along grain boundaries introduces mechanical weak spots that are responsible for the decline in Li-dendrite tolerance. Furthermore, the complex phase composition with several secondary phases creates nonuniform current distributions, which causes higher overpotentials (∼40 mV) during cycling, triggering Li nucleation and, thus, early cell failure.

## 3 Conclusions

This work demonstrates that atomic layer deposition of Al_2_O_3_ on LLZTO powders enables precise engineering of Li–Al–O interphases, significantly influencing sintering behavior, phase evolution, and electrochemical performance. Lithium diffusion during deposition transforms the amorphous ALD layer into a compositionally graded interphase, which further transforms into crystalline LiAlO_2_ over the course of high-temperature sintering. Thin and medium-thickness coatings (10–50 ALD cycles) effectively promote densification and uniform grain growth, leading to optimal microstructures and enhanced ionic conductivity. In contrast, excessive layer deposition (100 cycles) results in secondary phase accumulation (LiAlO_2_, Li_2_ZrO_3_, LaAlO_3_) and microstructural inhomogeneity, degrading electrochemical performance. The best balance between phase stability, microstructure, and electrochemistry was achieved with 25 ALD cycles (∼6.8 nm), exhibiting an ionic conductivity of 0.39 mS cm^–1^ and a CCD of 0.35 mA cm^–2^ while maintaining long-term cycling stability. Overall, this study provides mechanistic insight into ALD-enabled interfacial design in garnet-type SSEs and establishes thickness-dependent regimes for the sintering process which can act as guideline for optimizing coating thickness to improve the performance of all-solid-state lithium batteries.

## 4 Experimental Section

### 4.1 Powder Atomic Layer Deposition

Commercial Ampcera Li_6.4_La_3_Zr_1.4_Ta_0.6_O_12_ powder (pass 325 mesh, D50 ∼ 5 μm) was purchased from MSE Supplies LLC and coated in a Picosun R-200 Advanced ALD-reactor (Applied Materials, Inc.) at Nanexa AB, Sweden. The application of Al_2_O_3_ coatings was carried out at 225 °C by employing trimethylaluminum (EpiValence) and deionized water as precursors for self-limiting vapor-phase half reactions, with nitrogen utilized as the inert carrier gas (flow: 250 sccm). Prior to deposition, a stabilization period of 90 min was integrated to acquire a homogeneous temperature distribution throughout the sample (referred to as the pristine sample). Subsequent depositions were carried out in stop-flow mode to increase coverage of the complex sample geometry and for higher precision in layer thickness control. For each ALD subcycle, 20 time 1 s pulses of precursor, each followed by 30 s soaking time and nitrogen purging, were conducted. Single-crystal silicon (100) wafers were simultaneously introduced into the ALD-reactor during depositions as references.

### 4.2 High-Temperature Sintering

After deposition, LLZTO powders were pressed into cylinders (4 g each, *Ø* = 12 mm) by applying a force of 5 kN with a uniaxial press. The cylinders were placed into an alumina crucible with MgO inlet and lid. Small amounts of mother powder were placed below and on top to avoid interdiffusion from the crucible. Samples were sintered in air at a temperature of 1250 °C for 75 min (heating rate: 5 K/min) and naturally cooled down. Once the material was cold enough for transport, samples were immediately stored in an argon-filled glovebox. Thin pellets (0.7–0.8 mm) were cut using a low-speed diamond saw.

### 4.3 Material Characterization

For STEM analysis, samples were prepared as follows: powders were dispersed in semiconductor-grade isopropanol in an ultrasonic disc bath and one droplet (10 μL) was then applied onto a lacey carbon on 200 Ni-mesh. For the grain interface, a plane view lamella was prepared assuring the Gallium ion-beam is perpendicular to the grain boundary. The preparation was conducted in a Helios Nanolab 460F1 ^68^ at 30 kV for lift-out and milling to a U-shape lamella with final thickness ≤100 nm. Finally, the lamella was cleaned from front- and backside under ±5° inclination for 1 min at 5 kV and 5 min at 2 kV. STEM investigations were performed using a FEI Titan G2 80-200 CREWLEY system.^69^ Therefore, the device was operated at 200 kV, spot 6, and a condenser aperture of 70 μm was used, resulting in a convergence semiangle of 24.7 mrad. The acceptance angle of the BF was 10 mrad by setting the camera length to 65 mm. EDS mappings were obtained in an energy range of 0–20 keV. Fast Fourier transform and layer thickness analyses (accuracy: ∼1 nm) were carried out by using Velox software (Thermo Fisher Scientific Inc.).

The reference layer thickness of the ALD coating on a planar Si wafer was determined using spectroscopic ellipsometry (FS-8, Film Sense LLC). The obtained data including the ratio of the amplitudes of the reflected p- and s-polarized light components Ψ and their phase difference Δ with respect to wavelengths between 451 and 951 nm were fitted using a Cauchy model for the Al_2_O_3_ layer and a Herzinger model for the Si substrate including SiO_2_ as a native oxide layer to obtain layer thickness and refractive index. The resulting accuracy for the thickness lies below 1 Å and an average refractive index of 1.61 ± 0.01 was calculated.

Solid-state MAS NMR measurements were conducted using a Bruker AVANCE NEO spectrometer (18.78 T) equipped with a 3.2 mm triple-resonance H/X/Y CPMAS probe. ^27^Al spectra were recorded at operating frequencies of 208 MHz. MAS frequency was set to 16 kHz while sample temperature was maintained at 25 °C for all experiments and a 90° excitation pulse with a duration of 2.0 μs was used. NMR data were processed using TopSpin 4.1.0, including phase and baseline corrections. ^27^Al NMR spectra were externally referenced to AlF_3_ at −17 ppm.^70^

XPS on ALD-coated powder samples was carried out in an ultrahigh vacuum chamber with a typical base pressure of 2 × 10^–9^ mbar by nanoAnalytics GmbH, Germany. A monochromatic Al Kα X-ray source (*h*ν = 1486.6 eV) along with a high-resolution hemispherical electron analyzer (NEXSA G2, Thermo Fisher Scientific Inc.) was employed. The applied X-ray spot diameter on the sample amounted to 400 μm. For all samples, charge compensation was achieved by a combination of an electron flood gun (0.2 eV at 100 μA) and an argon ion source. High-resolution scans were recorded using a pass energy of 30 eV for all elements. The XPS processing software CasaXPS was employed to carry out peak fitting of C 1s HR-scans by minimizing the particular root-mean-square. A Shirley background was subtracted from all spectra prior to peak fitting. Equal full width at half maxima and a Voigt-like LA­(1,1,627) line shape were assumed for all peaks. The LLZTO samples were fitted with up to five peaks assigned to C–C/C–H, C–O, C=O/C–O–C, O–C=O, and Li carbonate species. The relative binding energies of the C–O and C=O/C–O–C peaks with respect to the C–C/C–H peak were allowed to vary within ±0.1 eV from 1.5 and 2.9 eV, respectively. Comparison of Al 2p HR-scans was facilitated by prior binding energy correction, i.e. by setting the binding energies of the C 1s C–C/C–H species to 284.8 eV for all samples.

Quantitative phase analysis was derived from powder XRD measurements using an EMPYREAN X-ray diffractometer (PANalytical). Sintered LLZTO pellets were ground into powders, filled into capillaries (*Ø* = 0.5 mm, *t* = 0.01 mm), and measured with Mo Kα radiation (50 kV, 40 mA) in transmission mode between 5 and 50° with 0.0084° as step size. The software package Diffrac.Topas Version 7 by Bruker was used for quantification through calculation of strain, lattice parameters and crystal size by the Pawley method. Occupancies, scale factors and Debye–Waller factors were refined via Rietveld refinement analysis.

The relative densities of sintered LLZTO pellets were derived from Archimedes’ measurements in which ethylene glycol was utilized as the liquid medium. The theoretical density necessary to calculate the relative density was derived using rule of mixtures based on XRD results.

Correlative SEM and EDS measurements were conducted using a Zeiss GeminiSEM 560 with an Ultim Max detector (100 mm^2^) (Oxford Instruments) for EDS measurements. EDS mapping was performed with an accelerating voltage of 10 kV at a working distance of 8.5 mm. The step size was set to 0.18 μm. EDS data were evaluated using Aztec software (Oxford Instruments). Samples were polished by using a JEOL Cross Section Polisher.

### 4.4 Electrochemical Tests

Electrochemical impedance spectroscopy was performed to determine ionic conductivity and activation energy of sintered LLZTO pellets using a Novocontrol broadband dielectric/impedance spectrometer equipped with an Alpha-A high performance frequency analyzer and a Quattro Cryosystem (temperature range: −100 to +100 °C). The AC voltage was set to 1.2 mV_rms_ and frequencies ranging from 20 MHz to 1 Hz were applied. Pellets were polished using SiC sandpaper (up to 4000 grit), followed by sputtering of 100 nm Au on both sides, acting as blocking electrodes. Data analysis was carried out using Zview software.

Chronoamperometry measurements were carried out at 25 °C using a BioLogic VSP-300 potentiostat. Polished LLZTO pellets (200 nm Au on each side) were assembled into a Swagelok cell and, subsequently, voltages of 0.25, 0.5, and 0.75 V were applied for 5 h each.

Li||LLZTO||Li symmetrical cells were assembled to investigate CCDs and long-term stability in plating/stripping tests. Therefore, polished (SiC 4000 grit) LLZTO pellets were deposited with 50 nm Au on each side and thin Li-sheets (*Ø* 8 mm) were welded onto the pellet. Afterward, pellets were placed between two stainless steel current collectors and heated up to *T*_set_ = 250 °C. As soon as the set-point was reached, the heating plate was turned down, and the assembly was placed at 60 °C into a Swagelok cell. Cell testing was performed using a multichannel potentiostat (VMP3, BioLogic) at 25 °C (Binder climate chamber). For CCD-measurements, the current density started at 0.05 mA cm^–2^ and increased in 0.05 mA cm^–2^ steps every five plating/stripping cycles (1 h each). Long-term experiments were conducted with a current density of 0.15 mA cm^–2^ with plating/stripping durations of 1 h each.

## Supplementary Material



## Data Availability

The data is available under CC-BY 4.0 license at 10.26165/JUELICH-DATA/1XFQBW.

## Notes

Additional experimental material, including FFT analysis, ellipsometry, HR-STEM–EDS, XPS, and SEM–EDS data, as well as Arrhenius plots (PDF)

## Abbreviations

LLZTO: Li_6.4_La_3_Zr_1.4_Ta_0.6_O_12_
SSE: solid-state electrolyte
ALD: atomic layer deposition
HR: high-resolution
STEM: scanning transmission electron microscopy
NMR: nuclear magnetic resonance spectroscopy
XPS: X-ray photoelectron spectroscopy
BF: bright field
EDS: energy-dispersive X-ray spectroscopy
FFT: fast Fourier transform
MAS: magic angle spinning
XRD: X-ray diffraction
EIS: electrochemical impedance spectroscopy
CA: chronoamperometry
CCD: critical current density
