# Supplementary material for: Tailoring Li–Al–O Interphases in Garnet-Type Solid-State Electrolytes via Powder Atomic Layer Deposition
Source: ACS Appl Mater Interfaces. 2026 Mar 25;18(13):18955–68. doi: 10.1021/acsami.5c23254 (PMC13067231; doi:10.1021/acsami.5c23254)
Supplement: Supplementary file 1 [file am5c23254_si_001.pdf]

Supporting Information

Tailoring Li-Al-O Interphases in Garnet-Type Solid-State Electrolytes via Powder Atomic Layer Deposition

Michael K. Steinhoff [a,b]*, *Anna Domgans* [a,b], *Jehad Ahmed* [a,b], *Roland Schierholz* [a], *Davis Thomas Daniel* [a], *Nabi Aghdassi* [a], *Shicheng Yu* [a], *Hermann Tempel* [a] *and Rüdiger-A. Eichel* [a,b]

[a] Institute of Energy Technologies - Fundamental Electrochemistry (IET-1), Forschungszentrum Jülich, 52428 Jülich, Germany

[b] Material and Processes of Electrochemical Energy Storage and Conversion, RWTH Aachen University, 52074 Aachen, Germany

* m.steinhoff@fz-juelich.de

KEYWORDS Surface modification, ALD powder coatings, Li-Al-O interphase, multi-phase structure, garnet-type electrolyte, LLZTO

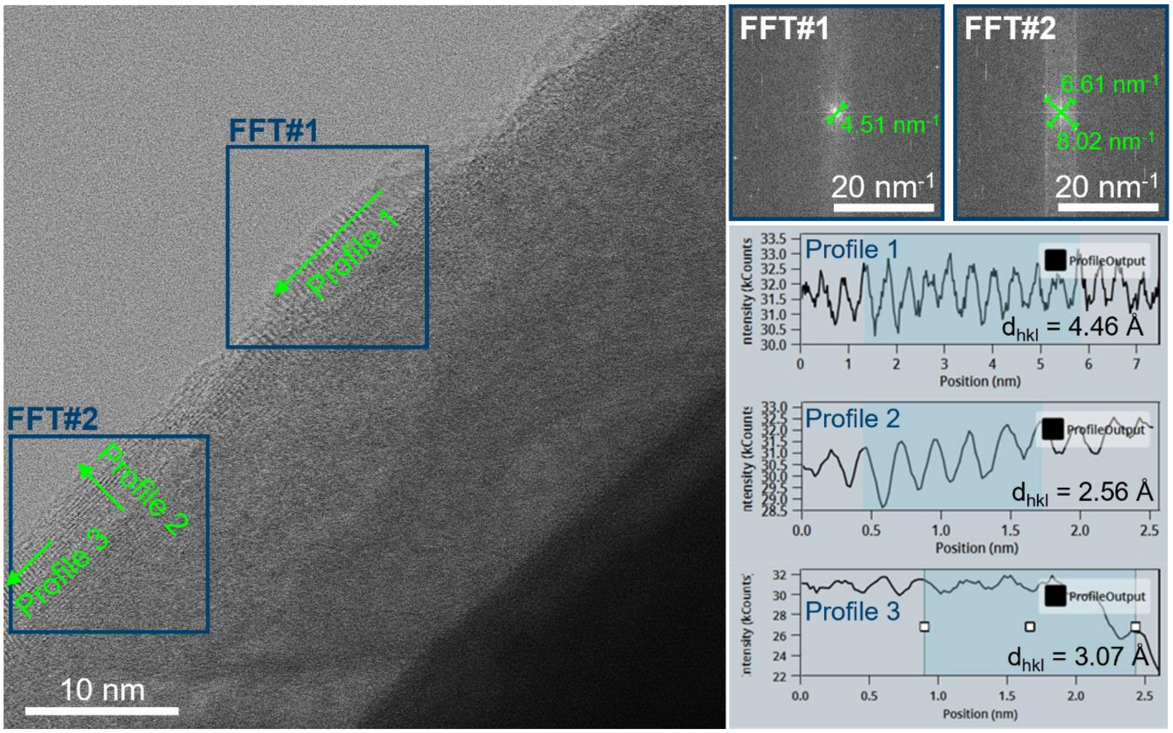

Figure S1. Fast Fourier Transform (FFT) analysis and line profiles on a high-resolution (HR) bright field (BF) scanning transmission electron microscopy (STEM) image of ALD-coated LLZTO (100 cycles) for lattice plane indexation.

Table S1. Lattice plane distances derived from FFTs and line profiles in **Figure S1**.

FFT#1		Profile 1		
$\|2g_1\|$ [nm^{-1}]	d_{hkl} [Å]	l [nm]	n	d_{hkl} [Å]
4.51	4.43	4.46	10	4.46

FFT#2		Profile 2		
$\|2g_2\|$ [nm^{-1}]	d_{hkl} [Å]	l [nm]	n	d_{hkl} [Å]
8.02	2.50	1.28	5	2.56

FFT#2		Profile 3		
$\|2g_3\|$ [nm^{-1}]	d_{hkl} [Å]	l [nm]	n	d_{hkl} [Å]
6.61	3.03	1.50	5	3.07

Table S2. Possible indexation of the lattice plane distances from **Figure S1** and **Table S1** with different aluminas and lithium aluminates.

Measured	α-Al_2O_3 ICSD# 9770		θ-Al_2O_3 ICSD# 82504		α-$LiAlO_2$ ICSD# 430184		α-$LiAl_5O_8$ ICSD# 10480		α-Li_5AlO_4 ICSD# 42697	
d_{hkl} [Å]	h k l	d_{hkl} [Å]	h k l	d_{hkl} [Å]	h k l	d_{hkl} [Å]	h k l	d_{hkl} [Å]	h k l	d_{hkl} [Å]
**4.46**	-	-	2 0 -1	4.53	-	-	1 1 1	4.57	0 0 2	4.56
									2 0 0	4.54
									0 2 0	4.47
**2.56**	1 0 4	2.55	1 1 -1	2.56	2 0 0	2.58	3 1 0	2.50	2 2 2	2.61
	1 1 0	2.38	1 1 1	2.45	2 0 1	2.39	3 1 1	2.38	3 0 2	2.52
									0 2 3	2.51
									2 3 0	2.49
									2 1 3	2.43
									3 1 2	2.43
									1 2 3	2.42
									3 2 1	2.42
									1 3 2	2.41
**3.07**	-	-	4 0 0	2.86	1 1 1	3.15	2 1 1	3.23	2 0 2	3.22
							2 2 0	2.80	0 2 2	3.19
									2 2 0	3.19
									2 1 2	3.03
									1 2 2	3.01
									2 2 1	3.01

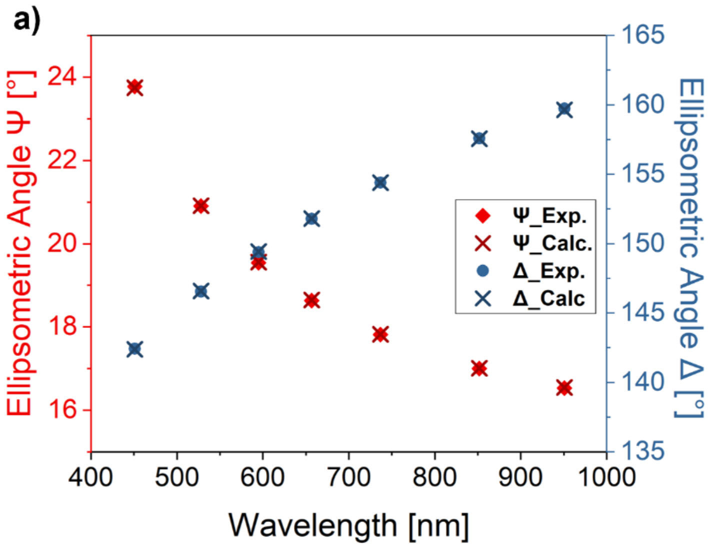

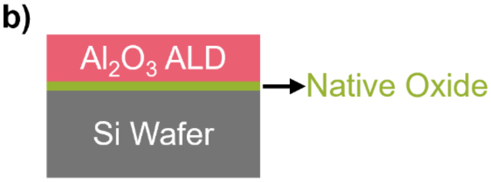

c)

100 ALD cycles	Layer Thickness [nm]
Spot 1	15.79
Spot 2	15.91
Spot 3	15.88

Figure S2. a) Ellipsometric angles Ψ and Δ vs. wavelength (raw data + fit) of the simultaneously coated planar Si wafer reference (100 ALD cycles); b) Schematic illustration of the multilayer model system including a thin native silicon oxide layer, which was used for fitting; c) Calculated film thicknesses for three different measurement points.

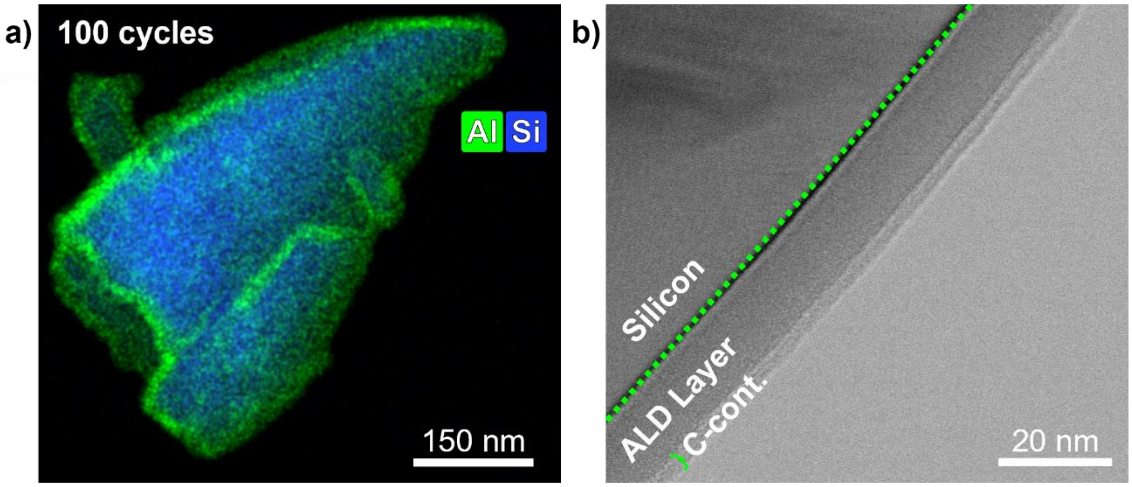

Figure S3. HR energy-dispersive X-ray spectroscopy (EDS) mapping (a) and BF-STEM image (b) of Si powder particles coated with 100 Al_2O_3 ALD cycles. The thin outer layer can be attributed to C-contamination from the microscope chamber. The O-signal was excluded to enhance clarity.

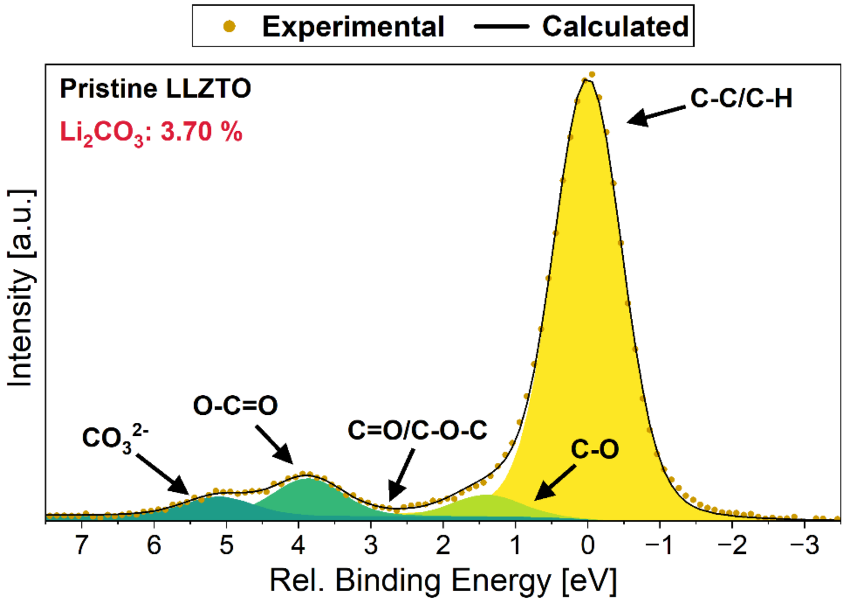

Figure S4. X-ray photoelectron spectroscopy analysis of the C 1s region. The spectrum was fitted with five peaks assigned to C-C/C-H, C-O, C=O/C-O-C, O-C=O, and lithium carbonate species. Relative binding energies were used with respect to the C-C/C-H signal.

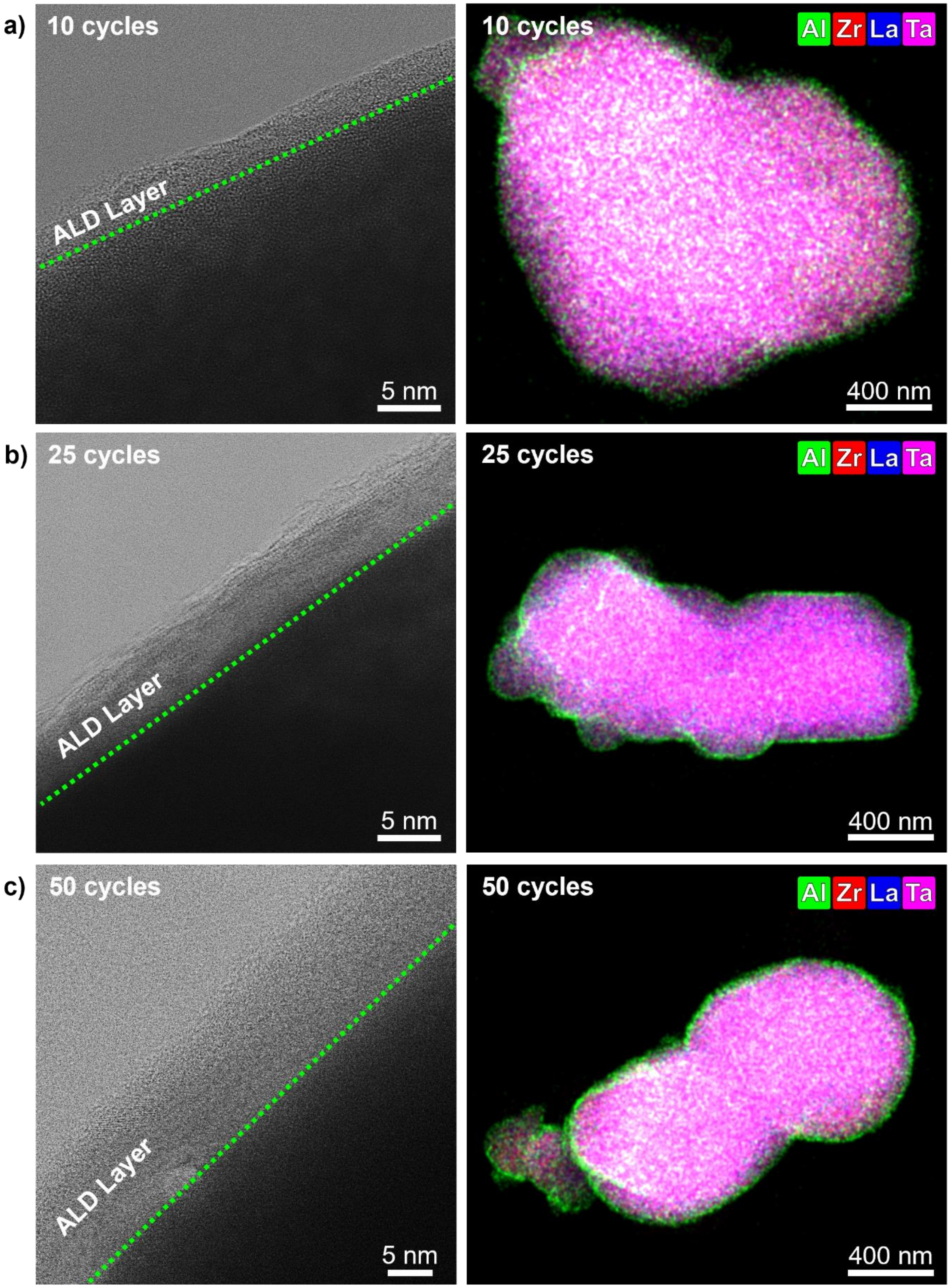

Figure S5. HR-BF STEM images and overview mappings of 10 (a), 25 (b), and 50 ALD cycles (c) coated on $Li_{6.4}La_3Zr_{1.4}Ta_{0.6}O_{12}$ (LLZTO) powder particles for layer thickness measurements.

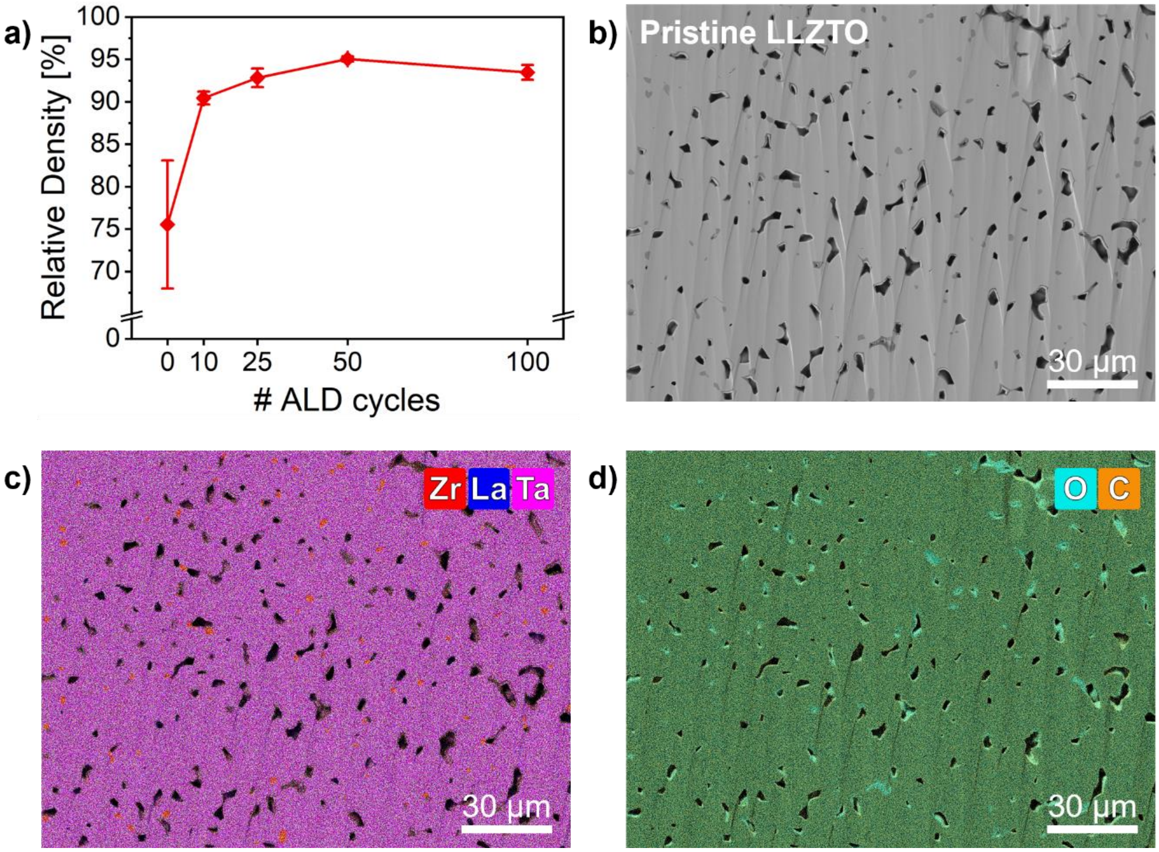

Figure S6. a) Relative densities obtained from Archimedes measurements on sintered pellets; b) Scanning electron microscopy image of a sintered pellet of pristine LLZTO; c) + d) EDS mappings on pristine LLZTO pellet in b).

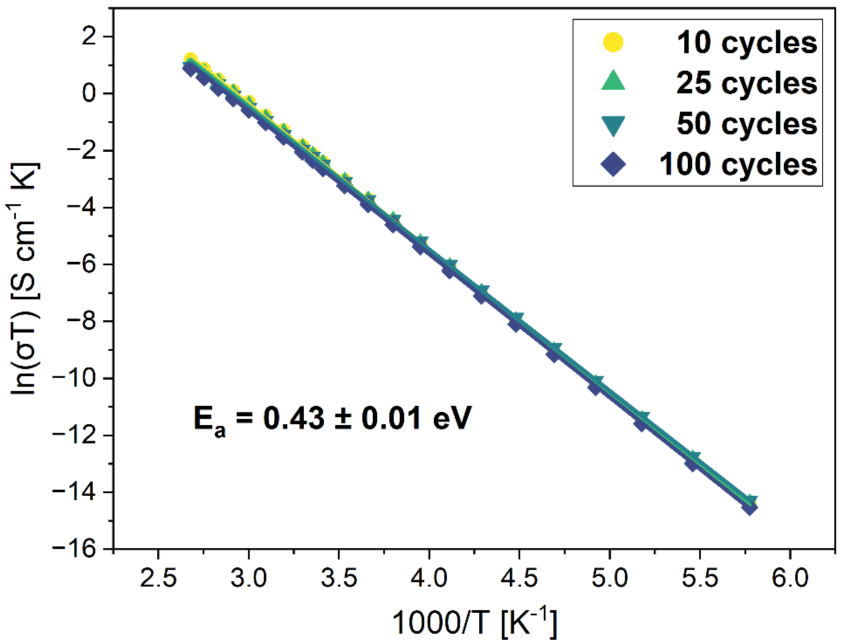

Figure S7. Arrhenius plots derived from EIS data between -100 and +100 °C.
